# Supplementary material for: Lentiviral single-cell MPRA of synthetic enhancers reveals motif affinity-based encoding of cell state specificity
Source: Genome Biol. 2026 Jul 14;27:308. doi: 10.1186/s13059-026-04186-9 (PMC13647567; doi:10.1186/s13059-026-04186-9)
Supplement: Supplementary file 1 [file 13059_2026_4186_MOESM1_ESM.docx]

**Additional File 1**

**Fig. S1 | Vector architecture minimizes promoter cross-talk in sc-lentiMPRA.** Schematic of the sc-lentiMPRA vector design comparing head-to-tail (a) and head-to-head (b) orientations of the Pol III–driven hU6 promoter and the Pol II–driven minimal promoter. Flow cytometry analysis of GFP expression for inert and CMV enhancer constructs promoter orientations. Head-to-tail arrangement of the hU6 and minimal promoter cassettes results in reduced cross-talk and improved independence between the two transcriptional units relative to the head-to-head configuration. pBC, Presence Barcode; minP, minimal Promoter; qBC, quantification Barcode.

**Fig. S2 | Vector architecture minimizes barcode swapping in sc-lentiMPRA. a Schematic illustration of barcode swapping introduced through lentiviral template switching during lentivirus production. b Experimental assessment of barcode pairings to quantify lentiviral recombination in K562 cells. Eight control constructs containing only the CMV promoter with different GFp and gRNA barcodes were cloned separately, but then pooled in equal amounts for lentiviral production. The fragment spanning the gRNA and GFP barcodes was amplified from genomic DNA and sequenced. The frequency of each barcode pairing across integrated constructs for the head-to-tail configuration is visualized in the heatmap. c** Schematic workflow used to determine recombination rates in Library Alpha HSPC screen. As in b, the integrated gRNA and GFP barcodes were amplified from genomic DNA and compared to the valid combinations from the barcode association library. **d** Experimental assessment of barcode swapping in HSPCs of the library Alpha screen. Swapping rate was quantified for each sequence separately and is shown as a histogram.

**Fig. S3 | Cloning Procedure and Barcode Association Libraries.** The complex enhancer library in the sc-lentiMPRA vector was cloned using a two-step PCR approach that introduced the gRNA scaffold, the minimal Promoter (minP), and two barcodes: the presence barcode (pBC) and the quantification barcode (qBC) (1). To map each enhancer to its corresponding pBC-qBC combination, fragments were amplified from the plasmid library (2) and sequenced using custom primers (3).

**Fig. S4 | CRS representation in plasmid libraries. a** Distribution of relative enhancer representation in plasmid library PoC, library Alpha, and library Beta after cloning. Each point indicates one element (putative enhancer) in the library and dotted lines visualize the 10^th^ and 90^th^ percentiles library elements. **b** Relationship between representation of the enhancers in library Alpha, and average activity measurements in sc-lentiMPRA (GFP UMIs/cell). **c** Same as b, but for library Beta. **d** Histograms depicting the number of distinct pBC-qBC barcode combinations per CRE. **e** Histograms depicting the number of cells in sc-lentiMPRA carrying the a pBC-qBC combination, relative to the total number of cells carrying the corresponding CRE.

**Fig. S5 | Flow cytometry sorting scheme for sc-lentiMPRA screens. a** After exclusion of debris, doublets, and dead cells, K562 cells were sorted for GFP-positive cells, which comprised 95.9% of parent population in the library PoC screen. Notably, the GFP gate was set immediately adjacent to the GFP-negative population in order to capture cells with low or repressive CRE activity. **b,c** Same gating strategy as in a, shown for primary HSPCs**.** After exclusion of debris, doublets, and dead cells, 48.2% were retained as live single cells. Of these, 14.7% were GFP-positive, corresponding to 7.1% of total events that were ultimately recovered and used for the screen. **d** Moderate correlation between the number of gRNAs observed and bulk MPRA activity stratified by TF/TF-pair of Lib Alpha and Lib Beta screen (Pearson´s R = 0.48). **e** Low correlations between the number of observations per CRE per cell state and the mean activity measured in sc-lentiMPRA (R = 0.14, Lib Alpha; R = 0.08, Lib Beta).

**Fig. S6 | UMI distributions of mRNA target capture.** Targeted gene capture in K562 cells and primary HSPCs shows strong enrichment of target genes relative to non-targeted transcripts.

**Fig. S7 | dCas9 abundance of K562 cell line and mouse primary HSPCs. a** Raw Ct values are shown for each primer set across K562, K562-dCas9, HSCs from. wild-type mice, HSCs expressing dCas9 samples, together with water controls. Dashed horizontal lines indicate the mean Ct of the water control for each primer. Different primers were used for the different Cas9 variant used in these lines. **b Cas9 abundance was normalized to the Actin controls. Overall, dCas9 signal was substantially higher in the K562 cells that in the mouse lines containing dCas9.**

**Fig. S8 | Effect of UMI-based filtering on barcode concordance and activity range in HSPCs.** **a** Fraction of barcode observations with a detected paired barcode as a function of increasing minimum UMI thresholds, shown separately for GFP-anchored and guide-anchored observations. For GFP-anchored observations, gfp0, gfp1, and gfp2 denote GFP-only preprocessing with minimum GFP UMI thresholds of >0, >1, and >2, respectively. For guide-anchored observations, guide0, guide1, and guide2 denote presence barcode-based preprocessing with guide UMI thresholds of >0, >1, and >2, respectively. For guide-anchored observations, we additionally show the fraction of matching (purple) or non-matching GFP barcodes (light purple) measured within the same cell (GFP UMI > 0), as well as the fraction of observations with no GFP detection at all (yellow). Increasing the guide-UMI threshold improves concordance between paired guide and GFP barcodes. **b** Mean enhancer activity, averaged across multiple synthetic sequences for each TF or TF pair in Library Alpha, shown for different preprocessing strategies and UMI thresholds. Lower thresholds are associated with a reduced activity range, suggesting increased noise if these measurements are taken into account.

**Fig. S9 | Correlations between total RNA UMI count, guide and GFP UMIs measured in K562s (library PoC). a** GFP UMIs show no significant correlation with total RNA UMI counts, indicating that enhancer activity measurements are not driven by overall transcriptional output or technical capture efficiency. **b** gRNA UMI counts display a weak but significant correlation with total RNA counts, suggesting a modest dependence of gRNA capture on overall technical capture efficiency. **c** GFP and gRNA UMI counts show no significant correlation, indicating minimal cross-talk between the Pol II–driven and Pol III–driven expression cassettes.

**Fig. S10 | Comparisons of sc-lentiMPRA preprocessing strategies and correlation with bulkMPRA data.** Data from Library PoC is shown for K562 cells, and data from Library alpha (see figure 2) is shown for HSPCs. **a** R² values between enhancer activity estimates derived from sc-lentiMPRA and bulk MPRA across different preprocessing strategies. GFP-only preprocessing quantifies enhancer activity directly from quantification (GFP) UMI counts, using a filter for minimum UMIs required of 0, 1 or 2 (gfp0, gfp1, gfp2). Presence barcode-based preprocessing first classifies cells as enhancer-positive based on presence-barcode (guide) detection thresholds (guide0, guide1, guide2 corresponding to presence barcode UMI > 0, >1, and >2, respectively), and subsequently quantifies enhancer activity using the corresponding GFP barcode counts. **b** Same analysis as in **a**, performed separately for two HSPC populations (CD55⁺ and CD55⁻).

**Fig. S11 | Sensitivity of sc-lentiMPRA in K562 cells and HSPCs.** Data from Library PoC is shown for K562 cells, and data from Library alpha (see figure 2) is shown for HSPCs. **a** Mean single-cell activity of inert random DNA control sequences, repressive Runx1 motif-containing sequences, and JeT promoter sequences in K562 cells. **b** Power analysis based on Wilcoxon rank tests in K562 cells showing the number of presence-barcode-positive single-cell measurements required to detect significant differences between JeT promoter activity sequences or repressive sequences and inert controls, respectively. Based on 100 pairwise Wilcoxon rank-sum tests with joint downsampling of all compared groups. **c Mean absolute GFP counts for various CRE sequences of Library PoC screened in K562 cells. In addition to Runx1 motif-containing sequences, the inert random DNA control sequences and the JeT promoter sequences, the plot includes 62 endogenous sequences that are differentially accessible in the HSPC differentiation landscape. d Mean single-cell activity of inert, repressive (Gfi1b-motif containing), and strong activator sequences (Trp53-motif containing) in HSPCs. e Power analysis in HSPCs, analogous to b, indicating the number of single-cell enhancer activity measurements required to detect significant activity differences between activator or repressive sequences and inert controls. NAs occur when both groups contain only zero values, resulting in no variance and preventing calculation of the Wilcoxon test statistic.**

**Fig. S12 | Marker gene expression of *in vitro* hematopoietic differentiation.** UMAP projection of the integrated single-cell dataset (Library Alpha, Library Beta) with cells colored by normalized expression of canonical markers spanning hematopoietic stem and progenitor states and downstream myeloid lineages. Shown are stem/progenitor markers (**Cd34, Kit, Mecom, Myb, Gata2),** granulocytic differentiation markers (**Mpo, Cebpe**), myeloid/monocytic markers (**Ctss, S100a9), a mast cell/basophil-associated marker (Mcpt8), erythroid markers (Hbb-bt, Kel), and a megakaryocyte-associated marker (Vwf**).

**Fig. S13 | Efficacy of enhancer measurements across hematopoietic cell states. a** Low-dimensional integrated representation of all cells (Library Alpha, Library Beta) colored by the number of distinct presence barcodes detected using UMAP. **b** Proportions of cells containing at least one presence barcode across all cell states, indicating efficient and even construct delivery, as well as minimal silencing across lineages in both experiments. **c** Distribution of CRE observations (number of cells/CRE) of the 84 candidate enhancers (Library Alpha), 80 candidate enhancers (Library Beta), respectively, per cell state. **d** Boxplot illustrating number of distinct pBCs observed per cell state and CRE of the 84 candidate enhancers (Library Alpha), and 80 candidate enhancers (Library Beta), respectively.

**Fig. S14 | Scaled enhancer activity across hematopoietic cell states.** Heatmap as in fig. 2d, showing only activity values for enhancers that were significantly different from random background DNA in a given cell state. Significance was determined by a Wilcoxon test, using a 0.1 FDR threshold. Other enhancer/cell state combinations are shown in grey.

**Fig. S15 | Transcriptome- and enhancer-based cell state clustering are highly concordant.** Tanglegram comparing hierarchical clustering of cell states based on transcriptome profiles and enhancer activity profiles. The overall dendrogram structures indicate that enhancer activity patterns recapitulate clustering of transcriptome-based cell states.

**Fig. S16 | Comparison between scMPRA data and a previous study of synthetic enhancers using bulk MPRA. 58 enhancers with identical sequence were covered in both studies. In Frömel et al., activity of these enhancers were measured in 7 FACS gates a** Single cell smart-seq2 data from Frömel et al (points in color) was projected on the scMPRA UMAP using Seurat integration. **b** Putative FACS gates corresponding to each cell from the scMPRA dataset were determined by majority voting within the cells’ local neighborhoods. scMPRA cells with no smart-seq2 cell in their local neighborhood were removed (grey). **c** Scatter plots comparing average RNA expression levels of key TFs per FACS gate between the two studies. Most TFs are highly correlated, Cebpa and Spi1 display lower correlations. **d** Correlation of enhancer activity across the two studies, stratified by cell state. **e** Correlation of enhancer specificity across the two studies, stratified by TF or TF combination. Cell states with low coverage in b,d were excluded.

**Fig. S17 | Cell state specificity of enhancers with the same design and different random DNA background. a** Correlation between replicate 1 and 3. A relatively coarse cell state clustering was chosen here to not bias correlation estimates by single-cell coverage). **b** Summary across TF and comparison to prior work. y axis shows mean (across CREs) of correlations (across cell states), stratified by TF. X axis shows activity correlations of design replicates from the bulk study (Frömel et al., 2025; PMID: 40345201).

**Fig. S18 | ROC curves of inert background control sequences.** ROC curves of fifteen different inert background control sequences demonstrate absence of cell state specificity.

**Fig. S19 |** **Trp53 enhancer activity across cell states. Relationship between sequence strength defined as the product of affinity and repeat number and** enhancer activity of synthetic Trp53 enhancers across hematopoietic cell states. Each point represents one enhancer design.

**Fig. S20 | Trp53 enhancer-expression relationships. a** Median per-subcluster coverage for all Trp53 and inert control sequences (n = 38 subclusters obtained from clustering single-cell gene expression data). Y axis refers to the median number of cells per enhancer across all Trp53/control enhancers. **b** Number of observations (guide-positive cells) per Trp53 or inert control sequence across subclusters. **c** Correlation between mean Trp53 expression and baseline activity (inert control sequences), averaged per subcluster. **d** Correlation between baseline activity and mean activity of low-affinity Trp53 enhancers, indicating that the observed Trp53 enhancer-expression relationship is not explained by baseline activity differences. **e** STRING interaction scores between Trp53 and a curated set of transcriptional coregulators. Factors significantly correlated with Trp53enhancer activity exhibit higher mean interaction scores with Trp53 compared to non-significant or negatively correlated factors. **f** Correlation between Trim25 expression and Trp53 enhancer activity across affinity classes, highlighting Trim25 as the strongest positively associated cofactor candidate.

**Fig. S21 | Relationships between TF expression and motif activity for all single-factor enhancers.** For all single-factor enhancers in library alpha, correlation of TF expression and enhancer activity was computed across 40 cell states. Different enhancers with the same repeat number and motif affinity, but different background sequences, were analyzed jointly.

**Fig. S22 | C/EBP enhancer activity across five affinity classes.** Enhancer activity of synthetic Cebpa enhancers across five affinity classes during early hematopoietic differentiation.

**Fig. S23 | Cebp enhancer-expression relationships. a** Median per-subcluster coverage for all Cebp sequences and inert control sequences (n = 45 subclusters). **b** Number of observations (guide-positive cells) per Cebpa or inert control sequence across subclusters. **c,d** Evidence for a non-linear relationship between Cebp factor expression and enhancer activity. **c.** Enhancer activity was modeled with a linear model of Cebpa + Cebpad + Cebpe expression, and the coefficient of determination (R^2^) was determined using 10-fold cross validation. Then, activity was modeled using project pursuit regression (PPR) of Cebpa + Cebpad + Cebpe expression, and R^2^ was quantified using 10-fold cross validation. In PPR, a linear combination of explanatory variables is passed through a non-linear smoothing function. Color scale indicates mean difference in R2 between the linear and the PPR model across 10 independent 10-fold cross validation schemes. *: sequences shown in d. **d** Example of non-linear relationship between Cebpd expression and mean activity of Cebp enhancer sequences.
